# Characteristics of Fresh Lumbar Spondylolysis Occurring below the Age of 9 Years

**DOI:** 10.31662/jmaj.2024-0039

**Published:** 2024-08-09

**Authors:** Ryo Himi, Tetsuya Ishikawa, Takaya Sugiyama, Kazuma Watanabe

**Affiliations:** 1 Department of Rehabilitation, Shizuoka Mirai Sports Orthopedics Clinic, Shizuoka, Japan; 2 Department of Orthopedic, Shizuoka Mirai Sports Orthopedics Clinic, Shizuoka, Japan

**Keywords:** Fresh lumbar spondylolysis, Elementary school students, Bone union, Advanced stage

## Abstract

**Introduction:**

This study aimed to compare two groups (9 years or younger [U-9] and 10 years or older [O-10]) of patients with fresh lumbar spondylolysis and elucidate their characteristics.

**Methods:**

This study enrolled 51 elementary school students diagnosed with fresh lumbar spondylolysis through magnetic resonance imaging between March 2015 and March 2022. Study 1 included 10 and 46 patients in the early- and late-grade groups, respectively. Patient characteristics at disease onset (sex, presence or absence of spina bifida occulta [SBO] in the affected vertebra, vertebral level, unilaterality or bilaterality of lesions, presence of a contralateral terminal stage, and disease stage) were compared between the two groups. Meanwhile, Study 2 included 34 patients with confirmed successful or failed bone union. The bone union rates in both groups were compared, and the factors affecting bone union in the entire study cohort were examined.

**Results:**

In Study 1, the proportions of SBO, bilateral, and advanced stage cases were significantly higher in the U-9 group than in the O-10 group. In Study 2, the bone union rate was significantly lower in the U-9 group than in the O-10 group.

**Conclusions:**

The proportions of SBO, bilateral, and advanced stage cases were significantly higher in the U-9 group than in the O-10 group. The bone union rate was significantly lower in the U-9 group than in the O-10 group.

## Introduction

Lumbar spondylolysis (LS) is a fatigue fracture that occurs during growth ^(1)^. The average age of onset has been reported to be approximately 14 years ^(2), (3), (4)^. Some studies that investigated LS in school students have reported no cases in children below 10 years old ^(3)^. LS is common among elementary school students, particularly those aged 6-12 years, with a reported prevalence rate of 9.3% ^(5), (6)^.

In elementary school students with LS, pseudarthrosis often progresses to spondylolisthesis ^(7)^; thus, bone union at this age is particularly desirable. LS is reportedly associated with spina bifida occulta (SBO) ^(6), (8), (9)^. SBO has been reported as one of the pathogenic factors ^(4)^ and an inhibitor of bone union ^(7), (9), (10)^. Common bone union inhibitors include the contralateral terminal stage ^(2), (11), (12)^, advanced stage ^(2)^, and bilateral cases ^(10), (13)^. There have also been studies that compared elementary school students with junior high school students and high school students ^(6)^. However, there is no study comparing elementary school students aged 9 years or younger (U-9) with those aged 10 years or older (O-10).

This study aimed to compare the U-9 group with the O-10 group and determine their characteristics. Two items were included in this study. Study 1 compared the characteristics of the U-9 and O-10 groups at disease onset, whereas Study 2 compared the bone union rate of the children.

## Materials and Methods

### Patient population

This study included 51 elementary school students who visited our clinic with lower back pain and diagnosed with fresh LS based on magnetic resonance imaging (MRI) short T1 inversion recovery image showing high signal intensity in the lumbar region from March 2015 to April 2024.

### Study design

**Figure 1.**
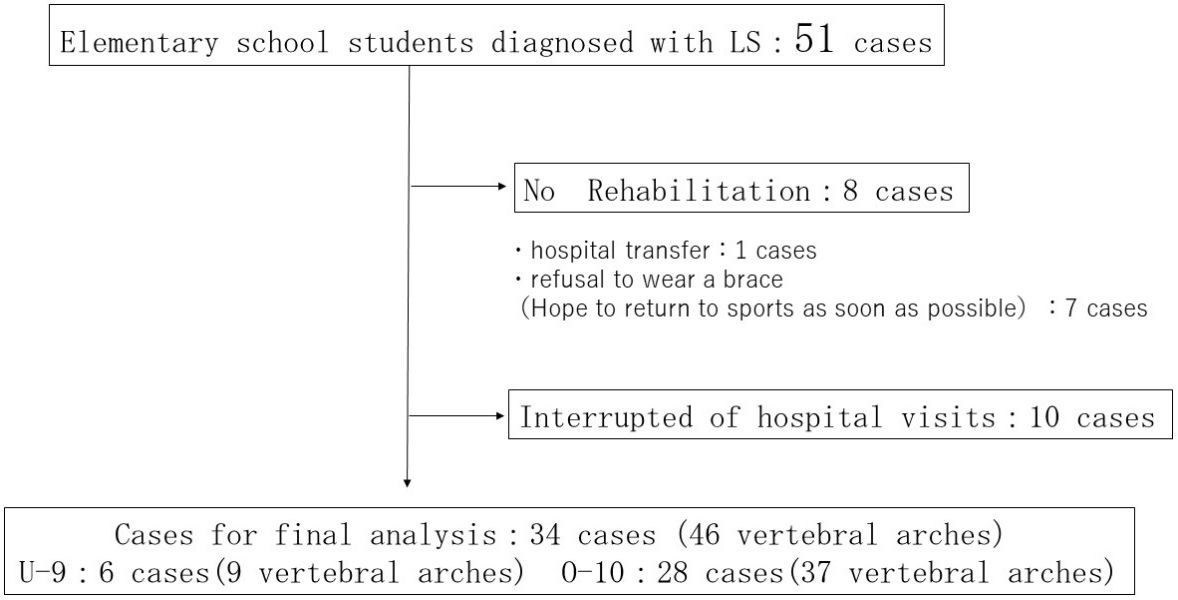
Exclusion criteria for cases with successful or unsuccessful bone union and cases for final analysis.

Study 1 included 51 cases: 10 cases (13 vertebral arches) in the U-9 group and 41 cases (53 vertebral arches) in the O-10 group. Sex, presence of SBO in the affected vertebra, vertebral body level, unilateral or bilateral cases, presence of contralateral terminal stage, and disease stage were compared between the groups. Study 2 (Figure 1) excluded 8 cases without rehabilitation and 10 interrupted of hospital visit from the total of 51 cases. A total of 34 cases were confirmed to bony union: 6 (9 vertebral arches) and 28 (37 vertebral arches) cases in the U-9 and O-10 groups, respectively. The bone union rate of the U-9 group was compared with that of the O-10 group.

### Statistical analysis

Fisher’s exact test was employed to compare the bone union rate of the U-9 group between Studies 1 and 2. The significance level was set to 5%.

### Classification by computed tomography (CT)

The disease stage was determined using horizontal and sagittal CT images. Early stage was defined as having no abnormal findings on CT or a finding of fissure on the sagittal section but not extending to the head.

Conversely, advanced stage was characterized by a sagittal section with a fissure extending from the caudal to the cephalic side and no bony continuity. The case was classified as terminal if the MRI did not show high signal in the lumbar interphalangeal region and the CT sagittal section showed a fissure extending from the caudal to the cephalad region with no bony continuity.

### Ethical consideration

The participants and their guardians, purpose of the study were informed orally and in writing, and their consent to data use was obtained by considering their personal information. We also ensured that the participants could refuse the use of their data through an opt-out procedure. The study was approved by the Ethics Review Committee of the Shizuoka Mirai Sports Orthopedic Clinic (approval number: 202441).

## Results

### Study 1 (Table 1 and 2)

No significant differences were observed in sex between the two groups: 9 men and 9 women in the U-9 group and 34 men and 7 women in the O-10 group. Furthermore, 8 (80.0%) of 10 patients in the U-9 group and 9 (22.0%) of 41 patients in the O-10 group had SBO on the affected vertebra. The proportion of patients with SBO was significantly higher in the U-9 group (*P* < 0.01). The vertebral body level was the fifth lumbar vertebra in 10 patients in the U-9 group, the fifth lumbar vertebra in 30 patients, and the other vertebra in 11 patients in the O-10 group. No significant differences were observed between the two groups at the vertebral body level. Regarding unilateral or bilateral cases, the U-9 group had 1 unilateral case and 9 bilateral cases, whereas the O-10 group had 22 unilateral cases and 19 bilateral cases. The proportion of bilateral cases was significantly higher in the U-9 group (*P* < 0.01). The number of patients with a contralateral terminal stage was 6 (50.0%) among 10 patients in the U-9 group and 7 (17%) among 41 patients in the O-10 group, showing no significant difference between the groups. At the disease stage, 5 and 8 vertebra arches were in the early and advanced stages, respectively, in the U-9 group, whereas 43 and 10 vertebra arches were in the early and advanced stages, respectively, in the O-10 group. The proportion of advanced stage patients was significantly higher in the U-9 group (*P* < 0.01).

**Table 1.** Characteristics of U-9 and O-10 Pathogenesis.

		U-9	O-10	*P*-value
		(10 cases)	(41 cases)	
- Sex	Male	9	34	1
	Female	1	7	
- SBO	(+)	8	9	0.001
	(−)	2	32	
- Vertebral body level	L5	10	30	0.09
	Other	0	11	
- Unilateral or bilateral	Unilateral	1	22	0.015
	Bilateral	9	19	
- Contralateral terminal stage	(+)	6	7	0.011
	(−)	4	34	

*P* < 0.01

**Table 2.** Comparison between U-9 and O-10 in Terms of Disease Stages.

		U-9	O-10	*P*-value
		(13 vertebral arches)	(53 vertebral arches)	
Disease stage	Early stage	5	43	0.004
	Advanced stage	8	10	

*P* < 0.01

### Study 2

The success or failure of bone union was confirmed in 34 patients and 46 vertebral arches. Excluded were cases with no rehabilitation (transfer, one case; early return to sports, seven cases) and nine cases were interrupted by hospital visits (Figure 1).

Successful bone union was confirmed in 4 of 10 arches (bone union rate, 44.4%) in the U-9 group and 30 of 37 arches (bone union rate, 81.1%) in the O-10 group. The U-9 group had a significantly lower bone union rate than the O-10 group (*P* < 0.05) (Table 3).

**Table 3.** Comparison of Bone Union Rates between U-9 and O-10.

		U-9	O-10	*P*-value
		(6 cases/9 vertebral arches)	(28 cases/37 vertebral arches)	
Bone union	(+)	4	30	0.039
	(−)	5	7	

*P* < 0.05

## Discussion

LS among elementary school students is occasionally observed in clinical practice. Nitta et al. reported that the prevalence of LS among elementary school students was 9.3% ^(5)^. Furthermore, Tsukagoshi et al. reported that elementary school students accounted for approximately 30% of all patients with LS ^(6)^, indicating that LS is common among this population. The bone union rate has been reported to be lower in elementary school students with LS. Its presence increases the risk of progression to lumbar spondylolisthesis; therefore, careful treatment is required. In general, the factors reported to inhibit bone union in LS include the presence of SBO ^(6), (8), (9)^, contralateral terminal stage ^(9), (10)^, advanced stage ^(2)^, and bilateral cases ^(11)^. In particular, elementary school students are more likely to have SBO and contralateral terminal stages than junior high school and high school students ^(6)^. SBO is a congenital osteogenesis imperfecta; therefore, some genetic factors are thought to influence the onset of LS in elementary school students ^(4)^.

In this study, the subjects were divided into the U-9 and O-10 groups based on the hypothesis that the difference between the groups would also affect the onset of LS. Age is related to disease onset. The U-9 group had significantly higher proportions of SBO, bilateral, and advanced stage cases than the O-10 group. Sakai et al. ^(4)^ reported that spondylolysis of the fifth lumbar vertebra in elementary school students is frequently identified as a terminal stage bone defect unrelated to a history of athletic activity. In this study, all cases in the U-9 group occurred in the fifth lumbar vertebra, and the SBO retention rate was 80%. Some cases of LS that developed in elementary school students, particularly in the early elementary school grade groups, were thought to be caused by genetic or congenital factors. As regards the etiology of LS, Sairyo et al. ^(14)^ reported that SBO is not a contributory factor to the development of LS. Contrarily, Tatsumura et al. ^(12)^ reported that a disruption of the bony ring structure causes overloading of the vertebral arch roots, and SBO is considered to be one of these bony ring structural disruptions. Therefore, SBO is presumed to be associated with LS. SBO, one of the genetic factors, may attract LS for the aforementioned reasons.

The reasons for the large number of bilateral and advanced stage cases in the U-9 group include the following: Elementary school students may not experience severe low back pain at the unilateral onset and may become aware of it at the contralateral onset ^(6)^. The development of the sense of touch, including the sense of pain, reaches the adult level, at least by the age of 9 years. It is inferred that neurodevelopmental immaturity may influence the time the patient becomes aware of the pain in the U-9 group. Even if a patient in the U-9 group develops unilateral LS, they do not experience severe low back pain; the disease is considered to gradually progress. If LS progresses to the unilateral side, the structural fragility of the vertebral arch may cause LS on the contralateral side ^(12)^. Thus, more advanced stage and bilateral cases are assumed to result in the U-9 group. In the early elementary school grade group, many cases had already progressed at the time of visit to a medical institution; therefore, they should be carefully treated. The bone union rate was significantly lower in the U-9 group than in the O-10 group. The presence or absence of SBO ^(8)^; stage progression, including contralateral pseudoarthrosis ^(2), (9), (10)^; and bilateral cases ^(10)^ have been reported to inhibit bone union in the entire elementary school population. In the present study, the proportions of SBO, bilateral, contralateral terminal stage, and advanced stage cases were higher in the U-9 group. As in previous studies, these factors may have counteracted bone union. Ishimoto et al. ^(15)^ reported that bone defects posterior and lateral to the vertebral arch interfere with fracture healing. As aforementioned, the presence of SBO and contralateral spondylolisthesis leads to disruption of the bony ring structure, which is associated with the development of LS, and the instability of this bony ring structure may counteract bone union after separation occurs.

We believe that the main treatment for LS in elementary school students is wearing a rigid trunk brace for the bone union. Fujimoto et al. ^(13)^ found that a rigid trunk brace can inhibit trunk extension, rotation, and lateral flexion. In a study ^(4)^ of patients with rigid trunk braces, the bone union rates were 100%, 93.8%, and 80.0% in the very early, early, and advanced stages, respectively, indicating good results. Among the elementary school students in this study, the bone union rate in the O-10 group was 80.5%, which is a good result compared with those in previous reports. However, the bone union rate of the U-9 group (36.4%) was significantly lower than that of the O-10 group. It is necessary to understand that the use of rigid trunk braces does not always lead to bone union among early elementary school students.

A limitation of this study is that Studies 1 and 2 are considered combined. We wish to increase the number of cases and revisit this study in the coming years.

## Article Information

### Conflicts of Interest

None

### Author Contributions

In accordance with CRediT (http://credit.niso.org), all authors on this study have contributed to Data curation, Formal analysis, Investigation, Methodology Validation, Writing original draft, Project administration, Supervision, Writing review & editing. All authors were involved in the preparation of the paper and reviewed the final manuscript.

### Approval by Institutional Review Board (IRB)

Shizuoka Mirai Sports Orthopedics Clinic ethics review committee

(approval number: 202441)
